# The effects of tislelizumab treatment on the health‐related quality of life of patients with advanced non‐small cell lung cancer

**DOI:** 10.1002/cam4.6361

**Published:** 2023-08-17

**Authors:** Dingzhi Huang, Caicun Zhou, Gisoo Barnes, Yiyuan Ma, Songzi Li, Lin Zhan, Boxiong Tang

**Affiliations:** ^1^ Department of Thoracic Medical Oncology, Lung Cancer Diagnosis and Treatment Centre, Key Laboratory of Cancer Prevention and Therapy Tianjin Medical University Cancer Institute and Hospital, National Clinical Research Centre for Cancer Tianjin China; ^2^ Department of Medical Oncology, Shanghai Pulmonary Hospital Tongji University School of Medicine Shanghai China; ^3^ Beigene Ltd California; ^4^ BeiGene (Beijing) Co., Ltd. Beijing China

**Keywords:** docetaxel, health‐related quality of life, non‐small cell lung cancer, patient‐reported outcomes, programmed cell death protein‐1 inhibitor

## Abstract

This study examined the health‐related quality of life (HRQoL) of patients with advanced non‐small cell lung cancer (NSCLC) receiving tislelizumab versus docetaxel in the open‐label, multicenter, Phase 3 trial called RATIONALE‐303 (NCT03358875). HRQoL was assessed with the EORTC QLQ‐C30, EORTC QLQ‐LC13, and the EQ‐5D‐5L instruments. A longitudinal analysis of covariance assessed the change from baseline to Week 12 and from baseline to Week 18. A time to deterioration analysis was also performed using the Kaplan–Meier method. Eight hundred and five patients were randomized to either tislelizumab (*n* = 535) or docetaxel, respectively (535 and 270 to tislelizumab and docetaxel, respectively). The tislelizumab arm improved while the docetaxel arm worsened in the QLQ‐C30 global health status/QoL scale score (difference LS mean change Week 18: 5.7 [95% CI: 2.38, 9.07, *p* = 0.0008]), fatigue (Week 12: ‐3.2 [95% CI: −5.95, −0.37, *p* < 0.0266]; Week 18: −4.9 [95% CI: −8.26, −1.61, *p* = 0.0037]), and QLQ‐LC13 symptom index score (Week 12: −5.5 [95% CI: −6.93, −4.04, *P* < 0.0001]; Week 18: −6.6 [95% CI: −8.25, −4.95, *p* < 0.0001]). The tislelizumab arm had improvements in coughing versus the docetaxel arm (Week 12: −4.7 [95% CI: −8.57, −0.78, *p* = 0.0188]; Week 18: −8.3 [95% CI: −13.02, −3.51, *p* = 0.0007]). The patients who received tislelizumab were less at risk for clinically meaningful worsening in the overall lung cancer symptom index scale (hazard ratio (HR): 0.24 [95% CI: 0.162, 0.356], *p* < 0.0001), dyspnea (HR: 0.74 [95% CI: 0.567, 0.958], *p* = 0.0109), coughing (HR: 0.74 [95% CI: 0.534, 1.019], *p* = 0.0309), and peripheral neuropathy (HR: 0.55 [95% CI: 0.370, 0.810] *p* = 0.0011). In general, tislelizumab versus docetaxel was associated with improved HRQoL and symptoms of lung cancer in patients who previously failed treatment with platinum‐containing chemotherapy.

## Graphical abstract

Patients with advanced non‐small cell lung cancer (NSCLC) have a poor prognosis and poor health‐related quality of life (HRQoL). This study found that patients treated with tislelizumab had greater improvements in the HRQoL measures of the EORTC QLQ‐C30 and EORTC lung cancer module (QLQ‐LC13) scales and scores compared with patients treated with docetaxel. These results support the favorable risk‐benefit ratio of tislelizumab as a second‐line therapy in patients with NSCLC who previously failed treatment with a platinum‐containing chemotherapy.

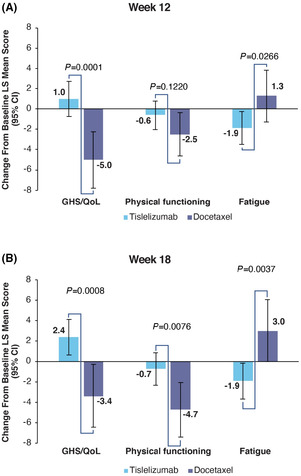

## 1 INTRODUCTION

Patients diagnosed with advanced non‐small cell lung cancer (NSCLC) at Stage IIIB and Stage IV have a relatively poor overall prognosis, with 5‐year survival rates of less than 5% and 2%, respectively.^1^ In patients with advanced NSCLC, there is a significant correlation between the disease and the presence of debilitating symptoms related to lung cancer, as well as poor health‐related quality of life (HRQoL).^2^, ^3^, ^4^, ^5^, ^6^, ^7^ Furthermore, an increase in symptom burden due to NSCLC disease progression and proximity to death leads to further deterioration of patients' HRQoL.^2^, ^4^, ^7^, ^8^ Particularly in patients with advanced NSCLC who have experienced disease progression after a platinum‐based regimen, a reduction or maintenance of patient‐reported lung cancer‐related symptoms are important goals of systemic therapies.^9^

Treatment with docetaxel, which has been considered the conventional approach of treating patients who have progressed after a platinum‐based regimen, has not produced significant benefits in HRQoL via reduction in symptom burden.^10^, ^11^ Clinical trials conducted recently on therapies that aim at the programmed cell death protein‐1 (PD‐1) and programmed death‐ligand 1 (PD‐L1) pathway, known as PD‐(L)1 inhibitors, have exhibited clinical benefit compared to docetaxel for patients who have advanced refractory NSCLC.^12^, ^13^, ^14^, ^15^, ^16^, ^17^ Along with clinical improvements, a reduction in some of the lung cancer‐related symptoms and improved HRQoL have also been reported in patients who received a PD‐(L)1 inhibitor as a second‐line therapy compared to those who were treated with docetaxel.^12^, ^18^, ^19^

Previous clinical studies have shown that patients with advanced squamous (sq‐) and non‐squamous (nsq‐) NSCLC who were administered tislelizumab in combination with chemotherapy have experienced clinical benefits compared to those who received chemotherapy alone.^20^, ^21^ With respect to HRQoL, the addition of tislelizumab to platinum‐based chemotherapy in a first‐line setting was associated with improvements in global health status and quality of life, relative to chemotherapy alone in patients with sq‐NSCLC and nsq‐NSCLC.^22^, ^23^ In patients with sq‐NSCLC, the combination of tislelizumab and platinum‐based chemotherapy has been linked with a decrease in lung cancer‐specific symptoms including coughing, dyspnea, and hemoptysis^23^; and for patients with nsq‐NSCLC, a reduction in symptoms such as coughing, chest pain, and dyspnea were reported.^22^

The RATIONALE 303 (NCT03358875) study was a Phase 3, randomized, open‐label, multicenter clinical trial that examined the effectiveness and safety of tislelizumab compared to docetaxel for NSCLC patients who had experienced disease progression after a previous treatment involving a platinum‐containing regimen.^24^ Compared with docetaxel, tislelizumab monotherapy prolonged overall survival (OS; median difference of 5.3 months), improved progression‐free survival (PFS; median 4.2 vs. 2.6 months; hazard ratio [HR]: 0.63 [95% confidence interval [CI]: 0.53–0.75]; *p* < 0.0001), and had a higher objective response rate (ORR; difference of 15.6%, 95% CI: 11.0–20.03; *p* < 0·0001).^24^ Furthermore, the rates of treatment‐emergent adverse events (TEAEs) were lower in the tislelizumab arm versus the docetaxel arm (≥ Grade 3 TEAE: 42.1% vs. 74.8%; Treatment Related ≥ Grade 3 TEAE; 15.7% vs. 66.3%), despite the median treatment duration being twice as long for the former.

The assessment of HRQoL using patient‐reported outcome (PRO) was a prespecified secondary objective in RATIONALE 303. The current analysis evaluated HRQoL and changes in patient‐reported lung cancer symptoms in patients treated with tislelizumab compared with docetaxel in patients with NSCLC who had experienced disease progression following a prior chemotherapy regimen containing platinum.

## 2 MATERIALS AND METHODS

### 2.1 Study design and population

Eligible patients from RATIONALE 303 were randomly assigned in a 2:1 ratio to receive either tislelizumab 200 mg every 3 weeks (Q3W) or docetaxel 75 mg/m^2^ Q3W until disease progression, intolerable toxicity, withdrawal of consent, or death.^24^ Patients in the tislelizumab arm were allowed to continue treatment beyond disease progression under protocol‐defined conditions, as deemed appropriate by the investigators.

Patients who were eligible for inclusion in the study were adults aged 18 years or older with locally advanced or metastatic sq‐ or nsq‐NSCLC, confirmed by histological analysis, regardless of tumor PD‐L1 expression, who experienced progressive disease during or after at least one platinum‐containing chemotherapy regimen (with no more than two prior systemic treatment lines) and had Eastern Cooperative Oncology Group (ECOG) performance status (PS) of 0 or 1. Patients who had treated, stable brain metastases were also included. Exclusion criteria included: prior docetaxel treatment for metastatic disease or prior immune checkpoint inhibitor treatment; known epidermal growth factor receptor (*EGFR*) mutation or anaplastic lymphoma kinase (*ALK*) translocation.

The study adhered to the ethical principles of the Declaration of Helsinki and Good Clinical Practice guidelines, as well as the requirements of the public registration of clinical trials and approved by the relevant Institutional Review Board or Independent Ethics Committee for each study site. Prior to participating in the study, written informed consent was obtained from all participants. Additional information on study design, efficacy, and safety data is reported elsewhere.^24^

### 2.2 Patient‐reported endpoints

The European Organization for Research and Treatment of Cancer (EORTC) Quality of Life Questionnaire Core 30 items (QLQ‐C30),^25^ the EORTC Quality of Life Questionnaire Lung Cancer 13 items (QLQ‐LC13)^26^ and the EuroQoL Five‐Dimensions Five‐Levels (EQ‐5D‐5L) questionnaire^27^ were used to assess HRQoL. Key PRO endpoints were the global health status/quality of life (GHS/QoL), physical functioning, and fatigue scales of the EORTC QLQ‐C30. Higher scores on the GHS/QoL scale and physical functioning scales indicated better outcomes, while higher scores on the fatigue scale indicated worse outcomes. For the QLQ‐LC13, the symptom index scale, dyspnea, coughing, peripheral neuropathy, pain in chest, pain in arm or shoulder, and hemoptysis items were used with higher scores indicating worse outcomes. The symptom index scale was calculated by taking the mean of all symptom scale scores. The Visual Analogue Scale (VAS) score of EQ‐5D‐5L recorded the patient's self‐rated health, where higher scores reflected better perceived health. These endpoints were selected due to their relevance to lung cancer and treatment‐related side effects, as well as their use in previous studies.^4^, ^5^, ^7^, ^12^, ^18^ The PROs were collected at baseline and on day 1 at every treatment cycle until to the end of treatment.

The key clinical cycles were week 12 (cycle 4) and week 18 (cycle 6) and were selected to measure change in PRO endpoints at the end of chemotherapy (week 12) and after chemotherapy treatment (week 18) close to the clinical scheduled visit for tumor assessment. At the start of each visit, patients were requested to answer the questionnaires before clinical assessments or intervention.

### 2.3 Statistical analyses

The PRO analyses consisted of all patients who were randomized and received at least one dose of the study drug and completed at least one assessment of HRQoL. The analysis included HRQoL assessments up to point of investigator‐assessed progressive disease (PD), as patients had the option to receive additional therapy following the assessment. If patients completed at least one item on a PRO instrument, they were deemed to have completed one HRQoL assessment. Compliance with the PRO assessments was determined as the proportion of questionnaires that were actually received out of the expected number (i.e., number of patients on treatment and are alive).

A longitudinal analysis of covariance was conducted to evaluate differences between the arms regarding mean score changes from baseline to week 12 and week 18 in the PRO endpoints. In line with the missing at random assumption, the response variable was the PRO score, while the covariates were treatment, study visit, treatment by study visit interaction, and randomization stratification factors (PD‐L1 expression in tumor cells and disease stage). Between‐group comparisons were reported as differences in the least square (LS) mean change from baseline with the 95% confidence interval (CI) and nominal *p*‐value. Changes from baseline that were between ±2 were considered to reflect maintenance, while a change of ±2 was considered improvement or worsening.

Time to deterioration (TTD) was examined using the QLQ‐LC13 symptom index scale, and the most relevant lung cancer symptoms of dyspnea, coughing, peripheral neuropathy, pain in chest, pain in arm or shoulder, and hemoptysis symptoms. TTD threshold was defined as time to first onset of ≥10‐points^28^ toward a worsening direction (e.g., increase score in symptom scales) from randomization to the first occurrence of a worsening score confirmed at the subsequent visit or death from any cause. The Kaplan–Meier method was used to estimate the deterioration curve in each group and the stratified log‐rank test was used to assess the treatment difference in TTD; one‐sided *p*‐value from stratified log‐rank test is presented. Efron's method of tie handling was used to assess between‐group differences. The stratification factors considered in the analysis included histology (squamous vs. non‐squamous), lines of therapy (second versus third), and PD‐L1 expression (≥25% vs. <25% tumor cells). Additionally, a descriptive analysis was performed for the EQ‐5D‐5L's VAS.

## 3 RESULTS

A total of 805 patients were randomly assigned to either the tislelizumab arm (*n* = 535) or the docetaxel arm (*n* = 270). The demographics and clinical characteristics were similar between the two treatment arms and were representative of the target patient population (Table 1). The data cutoff date for the present analysis was July 2021.

**TABLE 1:** Demographics and Baseline Characteristics.

	Tislelizumab (*n* = 535)	Docetaxel (*n* = 270)
Median age, years (range)	61.0 (28–88)	61.0 (32–81)
Patients aged < 65 years, *n* (%)	364 (68.0)	180 (66.7)
Sex, *n* (%)		
Male	416 (77.8)	206 (76.3)
Race, *n* (%)		
Asian	424 (79.3)	219 (81.1)
White	94 (17.6)	44 (16.3)
Other	17 (3.2)	7 (2.6)
ECOG performance status, *n* (%)		
0	115 (21.5)	50 (18.5)
1	420 (78.5)	220 (81.5)
Smoking status, *n* (%)		
Never	162 (30.3)	82 (30.4)
Current/former	373 (69.7)	188 (69.6)

*Note*: Table adapted from Zhou and colleagues (2023).^24^

Abbreviation: ECOG, Eastern Cooperative Oncology Group.

### 3.1 Compliance rates

The HRQoL analysis population included 789 patients: 533 in the tislelizumab arm and 256 in the docetaxel arm. At baseline, 530 (99.4%) of the 533 patients in the tislelizumab arm and 254 (99.2%) of the 256 patients in the docetaxel arm were compliant with the QLQ‐C30; at week 12, 368 (96.6%) of 381 and 109 (90.1%) of 121 patients, respectively, were compliant; at week 18, 318 (98.8%) of 322 and all 78 (100%) patients, respectively, were compliant (Table 2). There was similar compliance with the QLQ‐LC13 instrument.

**TABLE 2:** Compliance Rates for HRQoL Assessments.

	Tislelizumab (n = 533)	Docetaxel (n = 256)
	n (%)	n (%)
QLQ‐C30		
Baseline	530 (99.4)	254 (99.2)
Week 12	368/381 (96.6)	109/121 (90.1)
Week 18	318/322 (98.8)	78/78 (100.0)
QLQ‐LC13		
Baseline	530 (99.4)	254 (99.2)
Week 12	368/381 (96.6)	109/121 (90.1)
Week 18	318/322 (98.8)	78/78 (100.0)

Abbreviations: HRQoL, health‐related quality of life; QLQ‐C30, Quality of Life Questionnaire Core 30 items; QLQ‐LC13, Quality of Life Questionnaire Lung Cancer 13 items.

### 3.2 EORTC QLQ‐C30

**FIGURE 1:**
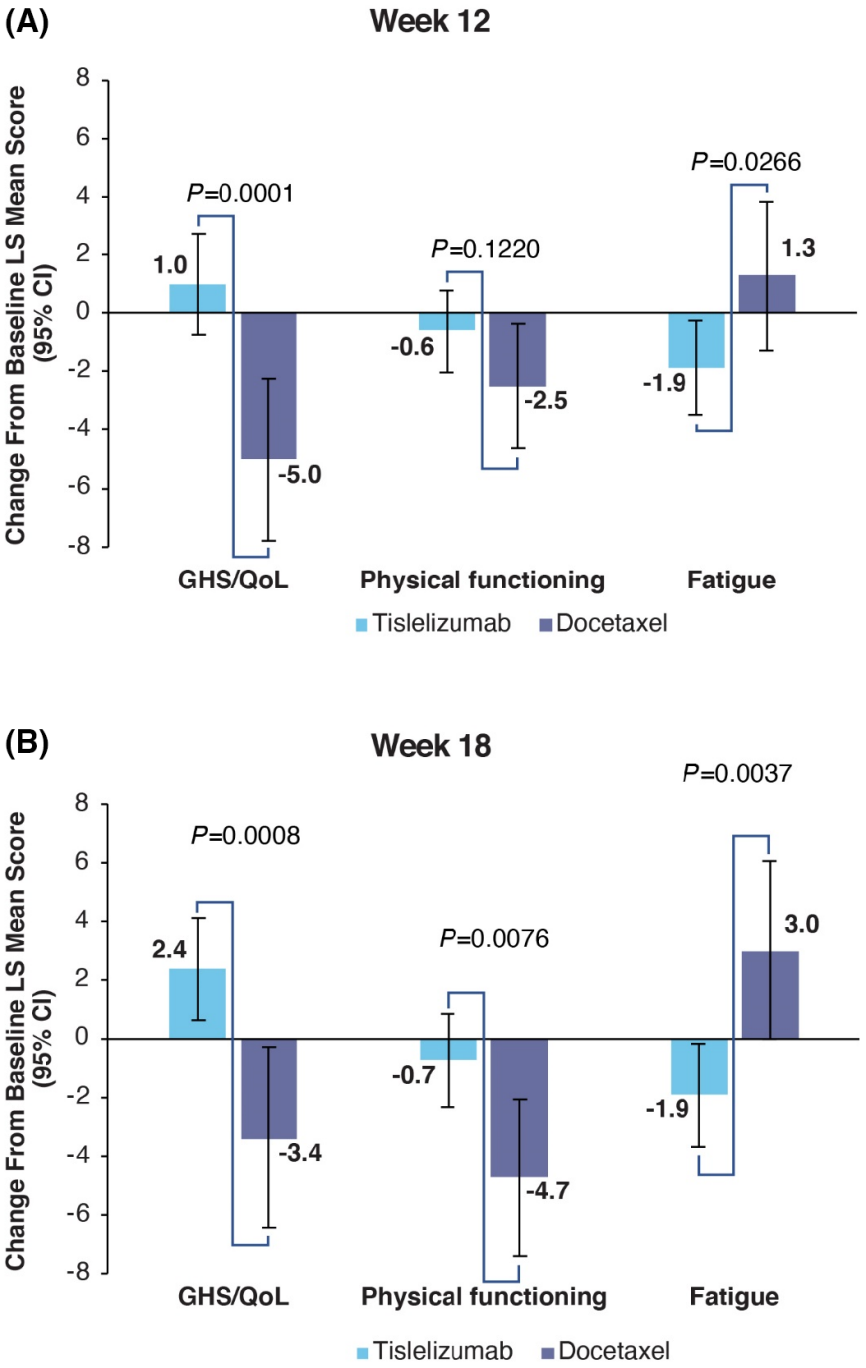
Change from Baseline for EORTC QLQ‐C30. CI, confidence interval; EORTC QLQ‐C30, European Organization for Treatment of Cancer Quality of Life Questionnaire Core 30 items; GHS/QOL, global health status/quality of life; LS, least squares.

Baseline means for the QLQ‐C30 are presented in Table S1. The GHS/QoL maintained at week 12 in the tislelizumab arm (LS mean change: 1.0 [95% CI: −0.76–2.68]) and worsened in the docetaxel arm (LS mean change: −5.0 [95% CI: −7.78 to −2.27]). At week 18, GHS/QoL improved in the tislelizumab arm (LS mean change: 2.4 [95% CI: 0.62–4.12]) and again worsened in the docetaxel arm (LS mean change: −3.4 [95% CI: −6.45 to −0.27]) (Figure 1A,B). The difference in change from baseline between the two arms was statistically significant at week 12 (difference in LS mean change: 6.0 [95% CI: 2.96–9.01, *p* = 0.0001]) and at week 18 (difference in LS mean change: 5.7 [95% CI: 2.38–9.07, *p* = 0.0008]).

In the tislelizumab arm, the physical functioning domain score maintained at week 12 (LS mean change: −0.6 [95% CI: −2.04 to 0.75]) and week 18 (LS mean change: −0.7 [95% CI: −2.32 to 0.82]), while worsening in the docetaxel arm at both week 12 (LS mean change: −2.5 [95% CI: −4.64 to −0.35]) and week 18 (LS mean change: −4.7 [95% CI: −7.42 to −2.06]). The difference in change from baseline between the two arms was not significant at week 12 (difference in LS mean change: 1.8 [95% CI: −0.50 to 4.19, *p =* 0.1220]) and statistically significant at week 18 (difference in LS mean change: 4.0 [95% CI: 1.07 to 6.92, *p =* 0.0076]).

The fatigue symptom scale score improved in the tislelizumab arm at both week 12 (LS mean change: −1.9 [95% CI: −3.50 to −0.26]) and week 18 (LS mean change: −1.9 [95% CI: −3.67 to −0.16]). In the docetaxel arm fatigue increased at week 12 (LS mean change: 1.3 [95% CI: −1.28 to 3.83]) and week 18 (3.0 [95% Cl: −0.04 to 6.07]). The difference in fatigue scores between the two arms was significant at both week 12 (difference in LS mean change: −3.2 [95% CI: −5.95 to −0.37, *p* = 0.0266]) and week 18 (difference in LS mean change: −4.9 [95% CI: −8.26 to −1.61, *p =* 0.0037]).

### 3.3 EORTC QLQ‐LC13

**FIGURE 2:**
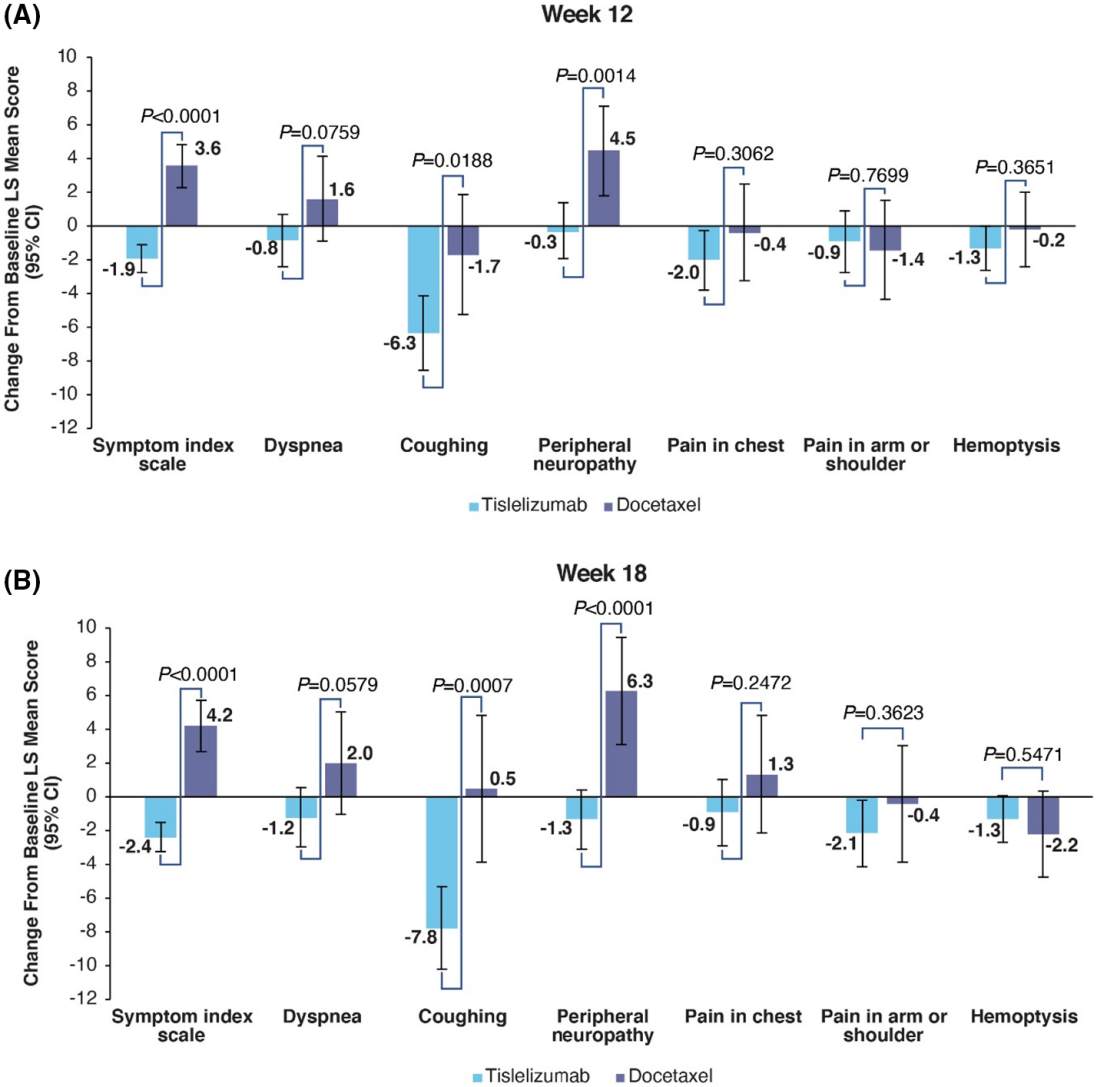
Change from Baseline for EORTC QLQ‐LC13. CI, confidence interval, EORTC QLQ‐LC13, European Organization for Research and Treatment of Cancer Quality of Life Questionnaire Lung Cancer 13 items; LS, least squares.

Baseline means for the QLQ‐LC13 are presented in Table S1. In the tislelizumab arm the symptom index scale score improved at week 12 (LS mean change: −1.9 [95% CI: −2.74 to −1.09]) and at week 18 (LS mean change: −2.4 [95% CI: −3.23 to −1.48]), while patients in the docetaxel arm experienced a worsening of the symptom index scale score at week 12 (LS mean change: 3.6 [95% CI: 2.27–4.88]) and week 18 (LS mean change: 4.2 [95% CI: 2.74–5.75]) (Figure 2A,B). The difference in change from baseline between the two arms was significant at both week 12 (difference in LS mean change: ‐5.5 [95% CI: −6.93 to −4.04, *p* < 0.0001]) and week 18 (difference in LS mean change: −6.6 [95% CI: −8.25 to −4.95, *p* < 0.0001]).

Dyspnea maintained in the tislelizumab arm at week 12 (LS mean change: −0.8 [95% CI: −2.42 to 0.73]) and improved at week 18 (LS mean change: −1.2 [95% CI: −2.93 to 0.56]). In the docetaxel arm dyspnea increased at both week 12 (LS mean change: 1.6 [95% CI: −0.87 to 4.16]) and week 18 (LS mean change: 2.0 [95% CI: −1.01 to 5.05]). The difference in change from baseline between the two arms was not significant at either week 12 (difference in LS mean change: −2.5 [95% CI: −5.24 to 0.26, *p* = 0.0759]) or at week 18 (difference in LS mean change: −3.2 [95% CI: −6.52 to 0.11, *p* = 0.0579]).

Coughing improved in the tislelizumab arm at week 12 (−6.3 [95% CI: −8.56 to −4.11]) and week 18 (−7.8 [95% CI: −10.23 to −5.30]). The docetaxel arm improved at week 12 (−1.7 [95% CI: −5.23 to 1.91]) and maintained at week 18 (0.5 [95% CI: −3.87 to 4.87]). The difference in change from baseline between the two arms was statistically significant at week 12 (−4.7 [95% CI: −8.57 to −0.78, *p* = 0.0188]) and at week 18 (−8.3 [95% CI: −13.02 to −3.51, *p* = 0.0007]).

Peripheral neuropathy maintained in the tislelizumab arm at week 12 (LS mean change: −0.3 [95% CI: −1.90 to 1.38]) and improved at week 18 (LS mean change: ‐1.3 [95% CI: −3.12 to 0.46]). In the docetaxel arm peripheral neuropathy worsened at week 12 (LS mean change: 4.5 [95% CI: 1.84–7.13]) and at week 18 (LS mean change: 6.3 [95% CI: 3.09–9.46]). The difference in change from baseline between the two arms was significant at both week 12 (difference in LS mean change: −4.7 [95% CI: −7.64 to −1.85, *p* = 0.0014]) and week 18 (difference in LS mean change: −7.6 [95% CI: −11.07 to −4.14, *p* < 0.0001]).

For pain in chest, the tislelizumab arm at improved at week 12 (LS mean change: −2.0 [95% CI: −3.79 to −0.24]) and maintained in the docetaxel arm (LS mean change: −0.4 [95% CI: −3.25 to 2.51]). At week 18, the tislelizumab arm maintained (LS mean change: −0.9 [95% CI: −2.86 to 1.06]) and worsened in in the docetaxel arm (LS mean change: 1.3 [95% CI: −2.15 to 4.84]).The difference in change from baseline between the two arms was not significant at week 12 (difference in LS mean change: −1.6 [95% CI: −4.80 to 1.51, *p* = 0.3062]) or week 18 (difference in LS mean change: −2.2 [95% CI: −6.05 to 1.56, *p* = 0.2472]).

For pain in the arm or shoulder, the tislelizumab arm maintained at week 12 (LS mean change: −0.9 [95% CI: −2.74 to 0.92]) and improved at week 18 (LS mean change: −2.1 [95% CI: −4.11 to −0.18]). The docetaxel arm slightly improved at week 12 (LS mean change: −1.4 [95% CI: −4.32 to 1.55]) and maintained at week 18 (LS mean change: −0.4 [95% CI: −3.86 to 3.06]).The difference in change from baseline between the two arms was not significant at week 12 (difference in LS mean change: 0.5 [95% CI: −2.73 to 3.69, *p* = 0.7699]) or week 18 (difference in LS mean change: −1.7 [95% CI: −5.51 to 2.02, *p* = 0.3623]).

For hemoptysis the tislelizumab arm improved at both week 12 (LS mean change: −1.3 [95% CI: −2.61 to −0.01]) and week 18 (LS mean change: −1.3 [95% CI: −2.69 to 0.05]). The docetaxel arm maintained at week 12 (LS mean change: −0.2 [95% CI: −2.39 to 2.03]) and improved at week 18 (LS mean change: −2.2 [95% CI: −4.73 to 0.37]).The difference in change from baseline between the two arms was not significant at week 12 (difference in LS mean change: −1.1 [95% CI: −3.58 to 1.32, *p* = 0.3651]) or week 18 (difference in LS mean change: 0.9 [95% CI: −1.93 to 3.64, *p* = 0.5471]).

### 3.4 EQ‐5D‐5L

Maintenance in the VAS score at weeks 12 and 18 were observed for the both the tislelizumab and docetaxel arms (Table 3).

**TABLE 3:** Change from Baseline for EQ‐5D‐5L VAS Scores at Week 12 and Week 18.

	Tislelizumab (*n* = 533)		Docetaxel (*n* = 256)	
	Observed Mean (SD)	Change from Baseline Mean (SD)	Observed Mean (SD)	Change from Baseline Mean (SD)
Baseline	79.4 (13.78)	–	76.8 (15.49)	–
Week 12	80.7 (13.78)	0.9 (11.68)	79.4 (12.93)	1.4 (11.93)
Week 18	81.4 (13.39)	1.0 (11.89)	78.2 (11.34)	1.7 (11.36)

Abbreviations: EQ‐5D‐5L, EuroQoL Five‐Dimensions Five‐Levels; SD, standard deviation; VAS, visual analogue scale.

### 3.5 Time to deterioration

The tislelizumab arm had a significantly lower risk of experiencing a deterioration event relative to the docetaxel arm for the QLQ‐C30 GHS/QoL score hazard ratio (HR: 0.77 [95% CI: 0.574–1.026], *p* = 0.00375), QLQ‐LC13 symptom index scale (HR: 0.24 [95% CI: 0.162–0.356], *p* < 0.0001), dyspnea (HR: 0.74 [95% CI: 0.567–0.958], *p* = 0.0109), coughing (HR: 0.74 [95% CI: 0.534–1.019], *p* = 0.0309), peripheral neuropathy (HR: 0.55 [95% CI: 0.370–0.810] *p* = 0.0011). Median TTD was not reached for hemoptysis and the pain in the symptoms **(**Table 4). The patients in both arms were in similar risk for hemoptysis, pain in chest, and pain in arm or shoulder.

**TABLE 4:** Time to Deterioration for GHS/QoL and the EORTC QLQ‐LC13.

	Tislelizumab (*n* = 533)	Docetaxel (*n* = 256)
GHS/QoL		
Patients with event, *n* (%)	150 (29.7)	70 (29.7)
Median time to deterioration, months (95% CI)	NE (NE, NE)	11.3 (4.99, NE)
Stratified^a^ HR (95% CI)	0.77 (0.574, 1.026)	
Stratified^a^ log‐rank test *p*‐value	0.0375	
Symptom index scale		
Patients with event, *n* (%)	50 (9.4)	60 (23.4)
Median time to deterioration, months (95% CI)	NR (NE, NE)	11.3 (6.93, NE)
Stratified^a^ HR (95% CI)	0.24 (0.162, 0.356)	
Stratified^a^ log‐rank test *p*‐value	<0.0001	
Dyspnea		
Patients with event, *n* (%)	176 (33.0)	87 (34.0)
Median time to deterioration, months (95% CI)	NE (20.96, NE)	4.9 (2.79, NE)
Stratified^a^ HR (95% CI)	0.74 (0.567, 0.958)	
Stratified^a^ log‐rank test *p*‐value	0.0109	
Coughing		
Patients with event, *n* (%)	118 (22.1)	59 (23.0)
Median time to deterioration, months (95% CI)	NE (NE, NE)	10.6 (5.65, NE)
Stratified^a^ HR (95% CI)	0.74 (0.534, 1.019)	
Stratified^a^ log‐rank test *p*‐value	0.0309	
Peripheral neuropathy		
Patients with event, *n* (%)	75 (14.1)	42 (16.4)
Median time to deterioration, months (95% CI)	NE (NE, NE)	NE (5.78, NE)
Stratified^a^ HR (95% CI)	0.55 (0.370, 0.810)	
Stratified^a^ log‐rank test *p*‐value	0.0011	
Pain in chest		
Patients with event, *n* (%)	94 (17.6)	38 (14.8)
Median time to deterioration, months (95% CI)	NE (NE, NE)	NE (10.58, NE)
Stratified^a^ HR (95% CI)	0.80 (0.543, 1.181)	
Stratified^a^ log‐rank test *p*‐value	0.1291	
Pain in arm or shoulder		
Patients with event, *n* (%)	115 (21.6)	31 (12.1)
Median time to deterioration, months (95% CI)	NE (NE, NE)	NE (NE, NE)
Stratified^a^ HR (95% CI)	1.27 (0.846, 1.906)	
Stratified^a^ log‐rank test *p*‐value	0.1261	
Hemoptysis		
Patients with event, *n* (%)	41 (7.7)	16 (6.3)
Median time to deterioration, months (95% CI)	NE (NE, NE)	NE (NE, NE)
Stratified^a^ HR (95% CI)	0.76 (0.420, 1.377)	
Stratified^a^ log‐rank test *p*‐value	0.1805	

Abbreviations: CI, confidence interval; EORTC QLQ‐LC13, European Organization for Research and Treatment of Cancer Quality of Life Questionnaire Lung Cancer 13 items; GHS/QOL, global health status/quality of life; HR, hazard ratio; NE, not estimated; NR, not reached.

^a^ Stratification factors: histology (squamous versus non‐squamous), lines of therapy (second versus third), and PD‐L1 expression (≥25% versus <25% tumor cells).

## 4 DISCUSSION

In RATIONALE 303, tislelizumab monotherapy demonstrated superior clinical efficacy compared to docetaxel in NSCLC patients who previously failed a platinum‐containing chemotherapy.^24^ Here we report that patients treated with tislelizumab also had more favorable HRQoL and a reduced lung cancer symptom burden, as assessed by the QLQ‐C30 and QLQ‐LC13, than patients who received docetaxel. Specifically, improvements were observed for the global health status and quality of life index scale in the tislelizumab arm relative to docetaxel at week 18. Physical functioning maintained in tislelizumab‐treated patients while worsening in the patients treated with docetaxel at both weeks. Fatigue symptoms decreased at week 12 and week 18 in patients treated with tislelizumab, while increasing at both weeks 12 and 18 in the docetaxel arm.

Overall lung cancer symptoms, as assessed by the QLQ‐LC13 symptom index score, improved at weeks 12 and 18 in the tislelizumab arm, while worsening at weeks 12 and 18 in the docetaxel arm. Improvements in coughing were significantly larger in tislelizumab‐treated patients relative to the docetaxel arm at both weeks 12 and 18. The tislelizumab arm also maintained (week 12) or slightly improved (week 18) in peripheral neuropathy while docetaxel patients worsened at both weeks 12 and 18. Time to deterioration analysis further showed that patients in the tislelizumab arm had a lower risk of experiencing clinically meaningful worsening in overall lung cancer symptom index scale, as well as for specific symptoms such as dyspnea, coughing, and peripheral neuropathy, over the course of treatment.

The findings of this study add to the expanding body of literature on the positive impact of PD‐(L)1 inhibitors on HRQoL of patients with advanced NSCLC.^12^, ^18^, ^19^ Similar to the current study, the Phase 3 CheckMate 017 study found significant improvements in the average symptom burden index of the Lung Cancer Symptom Scale associated with nivolumab compared to docetaxel, although the samples consisted of patients with sq‐NSCLC.^19^ In the Phase 2/3 KEYNOTE‐010 study, pembrolizumab was associated with improvements in the EORTC QLQ‐C30's GHS/QoL scale score relative to docetaxel, but unlike the current study these differences were nonsignificant.^12^ Similarly, the Phase 3 OAK study reported a nonsignificant improvement in the EORTC QLQ‐C30's GHS/QoL index scale score for atezolizumab as compared to docetaxel.^18^ Previous studies have also reported a decrease in overall lung cancer symptoms, including fatigue and coughing, which are consistent with the findings of the current study.^18^, ^19^ Finally, unlike several previous studies, TTD differences were not found for pain in chest, pain in arm or shoulder, and hemoptysis; however, significant differences in TDD were found in the tislelizumab arm in other lung cancer‐specific symptoms which were not observed in the previous studies.

The following limitations should be considered. First, an open‐label design was used and therefore patients were not blinded to treatment which could have impacted their responses to the PROs. Second, analysis did not investigate the relationship between PRO endpoints and clinical outcomes or adverse events. Previous studies have found that patients with disease progression experienced less worsening in HRQoL and lung cancer symptoms if treated with a PD‐(L)1 inhibitor compared to patients who received chemotherapy.^12^, ^18^ Moreover, a previous study of patients with NSCLC reported a 9% increase in survival with every 10‐point increase in global QOL as assessed by the EORTC QLQ‐C30.^29^ Taken together, these studies suggest that PROs assessing HRQoL and lung cancer symptoms may serve as a valid surrogate endpoints for diseases progression and overall survival. Though the tislelizumab arm experienced more improvements in HRQoL relative to the docetaxel arm, these changes did not reach clinical significance. Future analyses should consider additional stratification such as ethnic and/or regional differences endpoints, ECOG score, and/or other comorbidities within the current study population when possible. Finally, the missing at random assumption in the longitudinal analysis of covariance could be investigated. Future research will look at models that include reasons for treatment discontinuation and relationships to PRO outcomes.

Previous studies have found that when tislelizumab was added to platinum‐based chemotherapy as a first‐line treatment for sq‐NSCLC and nsq‐NSCLC, there were improvements in global health status, quality of life, and symptoms related to lung cancer.^22^, ^23^ The findings of the current study significantly extend tislelizumab's growing evidence as an agent that improves HRQoL and alleviates symptom burden to an even more at‐risk group of patients with NSCLC. In addition to tislelizumab's superior clinical benefits and safety profile compared to docetaxel,^24^ *patient‐reported outcomes provide additional evidence supporting the favorable risk–benefit ratio of* tislelizumab as a second‐line therapy for patients with NSCLC who had previously failed treatment with a platinum‐containing chemotherapy.

## AUTHOR CONTRIBUTIONS

**Dingzhi Huang:** Conceptualization (equal); data curation (equal); investigation (equal); methodology (equal); writing – review and editing (equal). **Caicun Zhou:** Conceptualization (equal); data curation (equal); investigation (equal); methodology (equal); supervision (equal); writing – review and editing (equal). **Gisoo Barnes:** Conceptualization (equal); data curation (equal); formal analysis (equal); supervision (equal); writing – original draft (equal); writing – review and editing (equal). **Yiyuan Ma:** Data curation (equal); formal analysis (equal); validation (equal); writing – review and editing (equal). **Songzi Li:** Data curation (equal); formal analysis (equal); supervision (equal); validation (equal); writing – review and editing (equal). **Lin Zhan:** Supervision (equal); writing – review and editing (equal). **Boxiong Tang:** Conceptualization (equal); methodology (equal); supervision (equal); writing – review and editing (equal).

## FUNDING INFORMATION

This study was sponsored by BeiGene, Ltd. Support for the development of this manuscript was provided by BeiGene, Ltd.

## CONFLICT OF INTEREST STATEMENT

GB, SL, LZ, and BT are employees of and own stock in BeiGene Ltd. DH reports no disclosures. YM is a former employee of Beigene and is now an employee of Boehringer International Ingelheim GmbH. CZ reports personal fees from Lily China, personal fees from BI, personal fees from Roche China, personal fees from MSD, personal fees from Qilu, personal fees from Hengrui, personal fees from Innovent Biologics, personal fees from C‐stone, personal fees from LUYE Pharma, personal fees from TopAlliance Biosciences Inc, personal fees from Amoy Diagnoistics, outside the submitted work.

## ETHICS STATEMENT

This protocol was approved by the ethics committees of the participating sites. The study was performed according to the ethical principles of the Declaration of Helsinki, Good Clinical Practice guidelines, and the requirements of the public registration of clinical trials. Written informed consent was obtained prior to participation in the study. The RATIONALE‐303 study is registered on clinicaltrials.gov as NCT03358875. Animal studies (N/A)

## Supporting information


Table S1

## Data Availability

All authors had access to the original data for the analyses described here. On request and subject to certain criteria, conditions, and exceptions, BeiGene will provide access to individual deidentified participant data from BeiGene‐sponsored global interventional clinical studies conducted for medicines (1) for indications that have been approved or (2) in programs that have been terminated. Data requests may be submitted to datadisclosure@beigene.com.
