# Supplementary material for: The effects of tislelizumab treatment on the health‐related quality of life of patients with advanced non‐small cell lung cancer
Source: Cancer Med. 2023 Aug 17;12(16):17403–12. doi: 10.1002/cam4.6361 (PMC10501279; doi:10.1002/cam4.6361)
Supplement: Supplementary file 1 — Table S1 [file CAM4-12-17403-s001.docx]

**Supplemental Table 1. Baseline Scores for the QLQ-C30 and QLQ-LC13**

|  | **Tislelizumab (N = 533)**  **mean, SD** | **Docetaxel  (N = 256)**  **mean, SD** |
| --- | --- | --- |
| **QLQ-C30** |  |  |
| GHS/QoL | 69.8 (18.92) | 69.1 (19.25) |
| Physical functioning | 86.6 (13.32) | 85.9 (14.74) |
| Fatigue | 21.0 (18.53) | 21.9 (18.56) |
| **QLQ-LC13** |  |  |
| Index Score | 11.9 (8.82) | 11.9 (10.14) |
| Dyspnea | 19.1 (14.70) | 20.4 (16.97) |
| Coughing | 31.3 (24.60) | 30.3 (25.23) |
| Peripheral neuropathy | 7.7 (18.36) | 5.8 (15.17) |
| Pain in chest | 14.9 (20.03) | 13.5 (22.30) |
| Pain in arm or shoulder | 13.3 (20.76) | 15.4 (25.42) |

CI, confidence interval; QLQ-C30, Quality of Life Questionnaire Core 30 items; QLC-LC13, Quality of Life Questionnaire Lung Cancer 13 items; GHS/QOL, global health status/quality of life.
